# Male sexual dysfunction associated with TNF-α inhibitors: an indication-stratified global pharmacovigilance analysis

**DOI:** 10.3389/fphar.2026.1915246

**Published:** 2026-09-14

**Authors:** Yi Chu, Min Luo, Wenyu Li, Xuehong Wang, Yuqian Zhou

**Affiliations:** Department of Gastroenterology, The Second Xiangya Hospital of Central South University, Changsha, Hunan, China

**Keywords:** adalimumab, etanercept, male sexual dysfunction, pharmacovigilance, TNF-alpha inhibitors

## Abstract

**Background:**

Tumor necrosis factor-α (TNF-α) inhibitors are widely used for chronic inflammatory diseases, but their effects on male sexual health remain uncertain. We investigated whether male sexual dysfunction (MSD) reporting varied across TNF-α inhibitors and clinical indications.

**Methods:**

We screened MSD reports associated with TNF-α inhibitors in WHO VigiAccess and performed a disproportionality analysis of individual case safety reports from the U.S. Food and Drug Administration Adverse Event Reporting System (FAERS) from 2004 to 2025. Four disproportionality algorithms were used to evaluate overall, indication-specific, and preferred term (PT)-specific reporting patterns. Time-to-onset was assessed using Weibull modeling.

**Results:**

No overall positive MSD signal was detected for any TNF-α inhibitor. In ankylosing spondylitis (AS), indication-level reporting odds ratio (ROR) signals were observed for adalimumab (ROR 1.80, 95% CI 1.11–2.92) and etanercept (ROR 2.21, 95% CI 1.37–3.55), but their direct comparison was not significant (ROR ratio 0.81, 95% CI 0.41–1.61). No positive PT-specific ROR signal was detected for either drug in AS. Among reports with usable onset dates, Weibull analysis suggested earlier reporting patterns.

**Conclusion:**

MSD reporting with TNF-α inhibitors may vary by clinical indication rather than represent a uniform class-wide pattern. The AS findings warrant cautious interpretation because both agents showed indication-level signals without a significant between-drug difference or positive PT-specific signals. These findings are hypothesis-generating and require prospective confirmation.

## Key points

- Real-world pharmacovigilance data indicate that male sexual dysfunction reporting with TNF-α inhibitors may vary across clinical indications rather than representing a uniform class-wide pattern.
- In ankylosing spondylitis, indication-level disproportionality signals were observed for both adalimumab and etanercept, while no statistically significant difference was found between the two agents and no positive PT-specific signals were detected.
- Time-to-onset analyses suggested an early reporting pattern among the limited subset of reports with usable onset dates; these findings should be interpreted as reporting-time patterns rather than estimates of clinical risk.

## 1 Introduction

Tumor necrosis factor-alpha (TNF-α) is a key proinflammatory cytokine in the pathogenesis of chronic autoimmune diseases, including ankylosing spondylitis (AS) (Yüksek et al., 2024), rheumatoid arthritis (RA) (Farrugia and Baron, 2016), psoriasis (Ps) (Li et al., 2019), and inflammatory bowel disease (IBD) (Souza et al., 2023). Consequently, TNF-α inhibitors are widely used to treat these conditions. While anti-TNF-α therapy is generally expected to improve sexual function by alleviating systemic inflammation, evidence regarding its specific effects on male sexual health remains limited and inconsistent. Most data are derived from small observational studies and isolated case reports describing potential treatment-emergent sexual dysfunction (Gao et al., 2023; Hidalgo-Rios et al., 2023; Ozge and Ugurlu, 2024; Bay et al., 2020; Nowowiejska et al., 2024).

A major challenge in evaluating this issue is that the underlying chronic diseases themselves are significant risk factors for sexual dysfunction (Nardone et al., 2025). For example, AS is strongly associated with erectile dysfunction (ED) (Zhang et al., 2023). Therefore, any observed signal for sexual dysfunction must be carefully assessed to separate the disease effect from the drug effect. Furthermore, TNF-α inhibitors are not structurally identical. For instance, etanercept is a receptor-fusion protein with a broader cytokine-binding spectrum, whereas adalimumab is a highly specific monoclonal antibody (Lim et al., 2018; Jang et al., 2021). These structural differences raise the possibility of heterogeneity in adverse-event reporting across individual TNF-α inhibitors, although the clinical significance of such differences remains uncertain.

Given the growing global use of TNF-α inhibitors, understanding variation in safety reporting across drugs and clinical indications is clinically important. Pharmacovigilance offers a practical real-world approach to detect such reporting patterns. Therefore, this study aimed to explore whether TNF-α inhibitors are associated with male sexual dysfunction (MSD) using two global pharmacovigilance databases: the U.S. Food and Drug Administration Adverse Event Reporting System (FAERS) and the World Health Organization (WHO) VigiAccess. By employing indication-specific stratification, we aimed to distinguish potential drug-related reporting patterns from disease-related confounding. These findings may improve understanding of indication-related heterogeneity in MSD reporting and inform the interpretation of pharmacovigilance signals in clinical practice.

## 2 Materials and methods

This study was designed, conducted, and reported in accordance with the Reporting recommendations for disproportionality analyses of spontaneous reporting systems in pharmacovigilance (READUS-PV) guidelines (Fusaroli et al., 2024).

### 2.1 Data processing and stratification

#### 2.1.1 Data sources

Data for this study were obtained from two global pharmacovigilance databases: the World Health Organization (WHO) VigiAccess (https://www.vigiaccess.org/) with reports current as of 7 June 2025; and the U.S. Food and Drug Administration Adverse Event Reporting System (FAERS) (https://fis.fda.gov/extensions/FPD-QDE-FAERS/FPD-QDE-FAERS.html), utilizing data files from January 2004 to March 2025. All analyses were performed using Microsoft Excel 2021 and R Studio (version 2025.09.1).

#### 2.1.2 Data modules and extraction

FAERS consists of seven core data modules. In the present study, the DEMO module was used to obtain case identifiers and demographic information; the DRUG module to identify drug exposure and role codes; the REAC module to identify adverse events; the INDI module to determine therapeutic indications; the THER module to calculate time-to-onset; the OUTC module to describe clinical outcomes; and the RPSR module to identify reporter type. Data standardization procedures strictly adhered to FDA guidelines for duplicate record removal and quality control.

Key Identifier Extraction: The fields PRIMARYID, CASEID, and FDA_DT (the date the report was received by FDA) were extracted from the DEMO dataset to serve as core identification fields.

Deduplication Strategy: Duplicate reports were managed through a two-step prioritization process: (1) when multiple records shared the same CASEID, only the most recent FDA_DT entry was retained; (2) if multiple records matched both CASEID and FDA_DT, the record with the highest PRIMARYID was preserved (USFaD, 2023).

#### 2.1.3 Drug and event coding

All reports involving the five TNF-α inhibitors relevant to this study were extracted, deduplicated, and coded. Adverse events were coded according to the Medical Dictionary for Regulatory Activities (MedDRA, version 27.0). The focus was specifically on male sexual dysfunction events, such as Erectile Dysfunction and Decreased Libido. A detailed list of the MedDRA preferred terms (PT) used is provided in Supplementary Table S1.

### 2.2 Case selection criteria

Within the FAERS database, each drug’s role in an adverse event is classified as Primary Suspect, Secondary Suspect, Concomitant, or Interacting. To enhance the specificity of signal detection, only Primary Suspect drugs were included in the primary analysis, as other roles entail greater uncertainty regarding causal attribution. Drug identification was based on both brand and generic names according to official FDA documentation.

In addition, a secondary analysis was performed to characterize Concomitant drugs co-administered with TNF-α inhibitors, stratified according to their pharmacological mechanisms and their potential to affect male sexual function.

Only male cases were retained for analysis because the study specifically focused on male sexual dysfunction as the adverse event of interest.

### 2.3 Concomitant medication classification

Concomitant medications were categorized based on their presumed mechanism of influence on male sexual function.

Direct-acting drugs were defined as agents known to directly induce or contribute to male sexual dysfunction, including beta-blockers (Corona et al., 2025), lipid-lowering agents (Adeeb et al., 2025), antidepressants (Montejo et al., 2019), H_2_-receptor antagonists (Park et al., 2006), drugs for benign prostatic hyperplasia (including 5-α-reductase inhibitors) (Corona et al., 2017), antipsychotics (Par et al., 2012; de Boer et al., 2015), anxiolytics, hypnotics and sedatives, and antiepileptics (including benzodiazepines) (Velurajah et al., 2022).

Indirect-acting drugs were defined as glucose-lowering agents used to treat diabetes mellitus. Because the FAERS database lacks explicit diagnostic information, the use of antidiabetic medications was employed as a proxy indicator of diabetes mellitus, representing patients whose metabolic and vascular alterations may indirectly contribute to sexual dysfunction rather than reflecting pharmacologic effects *per se* (Feng et al., 2024).

The “Both” category referred to patients who were concurrently using both direct-acting and indirect-acting medications. These classifications were used in secondary descriptive analyses to assess potential confounding by concomitant medications and underlying metabolic conditions.

### 2.4 Comparative framework

To compare the signal strength of TNF-α inhibitors, two reference drugs were selected for analysis: finasteride and omeprazole. Finasteride was chosen as a positive control, as it is well documented to induce male sexual dysfunction—including decreased libido, erectile dysfunction, and ejaculatory disorders (Traish, 2018). Omeprazole was used as a negative control due to its extensive clinical use and lack of established association with sexual dysfunction. Although isolated case reports have described erectile dysfunction events, omeprazole has not demonstrated a disproportional signal in pharmacovigilance databases, making it a suitable reference for comparative interpretation of the detected safety signals (Perry, 2021).

The disproportionality estimates derived for TNF-α inhibitors were therefore interpreted in comparison with these reference drugs to allow a clear and direct interpretation of the detected safety signals.

### 2.5 Disproportionality analysis methodology

Four disproportionality methods—Reporting Odds Ratio (ROR), Proportional Reporting Ratio (PRR), Empirical Bayes Geometric Mean (EBGM), and Bayesian Confidence Propagation Neural Network (BCPNN)—were employed to assess potential associations between drug exposure and adverse events (AEs) in the database (Bate and Evans, 2009).

For each method, a 2 × 2 contingency table (Supplementary Table S2) was constructed, where a represents the number of reports of the target event associated with the drug of interest, b represents the number of reports of all other (non-target) events for the same drug, and c and d represent the corresponding counts for all other drugs in the database. The variables a–d denote the cell counts in this contingency table, with their specific interpretations varying depending on the level of comparison (e.g., drug-level, PT-level, or indication-level analyses). Overall analysis: target event = any predefined MSD PT record; indication-specific analysis: target event = reports containing ≥1 predefined MSD PT within that indication; PT-specific analysis target event = one specified PT within that indication.

#### 2.5.1 Reporting odds ratio and inter-drug odds ratio (ID-OR)

The ROR was calculated as follows:

$ROR=\left(a\times d\right)\;/\;\left(b\times c\right)$ ; and its 95% confidence interval (CI) was computed as:

$$95\%\;\;CI=\exp\left[\ln\left(ROR\right)\pm \;1.96\times \surd \left(1/a+1/b+1/c+1/d\right)\right]$$

An ROR >1 indicates higher reporting disproportionality, whereas an ROR <1 indicates lower reporting disproportionality. A positive disproportionality signal was defined as a ≥3 and the lower limit of the 95% CI > 1.

To compare the disproportionality signal strength between two drugs under the same indication, the ratio of their RORs was calculated as: ROR_ratio=ROR_Drug1 / ROR_Drug2 .

Assuming independence between ROR estimates, the variance of the logarithmic ROR ratio was approximated as: $Var\left[\ln\left(ROR_{ratio}\right)\right]=Var\left[\ln\left(ROR_{Drug1}\right)\right]+Var\left[\ln\left(ROR_{Drug2}\right)\right]$ .

Accordingly, the 95% confidence interval (CI) was calculated as:

$$95\%\;\;CI=\exp\left[\ln\left(ROR\_ratio\;\right)\pm \;1.96\times \surd \left(Var\left[\ln\left(ROR\_Drug1\;\right)\;\right]+Var\left[\ln\left(ROR\_Drug2\;\right)\;\right]\;\right)\right]$$

When the 95% CIs of the two RORs overlapped or the CI of the ROR ratio included 1, the difference was interpreted as not statistically significant.

#### 2.5.2 Preferred term–or indication-specific ROR calculation

The same 2 × 2 contingency-table approach was applied to indication- and PT-specific analyses. For indication-specific analyses, reports were restricted to the corresponding indication stratum. For PT-specific analyses, each study drug–PT pair was compared with the same PT reported for all other drugs within the corresponding indication stratum. Thus, the comparator for the PT-specific analysis was not the other TNF-α inhibitor, but all other drugs reported within the same indication stratum. This approach enabled a more granular evaluation of drug–event reporting patterns while accounting for the underlying clinical indication.

#### 2.5.3 Other disproportionality methods

The same contingency table framework (Supplementary Table S2) was applied to PRR, EBGM, and BCPNN, which share a computational framework similar to that of ROR, and the detailed formulas have been presented in our previous work.

##### 2.5.3.1 Signal detection criteria

For ROR, a positive disproportionality signal was defined as a ≥3 and the lower limit of the 95% CI > 1.

For PRR, a positive disproportionality signal was defined as a ≥3 and the lower limit of the 95% CI > 1.

For BCPNN, a positive disproportionality signal was defined as a ≥3 and IC025 > 0.

For MGPS, a positive disproportionality signal was defined as a >0 and EBGM05 > 2.0.

### 2.6 Time-to-onset of adverse events

Time-to-onset (TTO) of AEs was analyzed using a Weibull distribution model, which characterizes the temporal pattern of event occurrence based on two parameters: the shape parameter (β) and the scale parameter (α). This approach enables characterization of whether the reporting pattern of an adverse event is concentrated earlier, remains approximately constant, or tends to occur later following drug exposure (Sauzet and Cornelius, 2022; Cornelius et al., 2012).

#### 2.6.1 Interpretation of parameters

Shape parameter (β): The β value determines the time-dependent behavior of the hazard function. β < 1 indicates a pattern in which reported events are more concentrated earlier after treatment initiation; β = 1 indicates an approximately constant reporting pattern over time; and β > 1 indicates a pattern in which reported events tend to occur later.

Scale parameter (α): The α value represents the characteristic time to onset—the time point at which approximately 63.2% of the modeled reported events are expected to have occurred. A larger α value suggests a generally longer time to onset or a wider distribution of onset times.

### 2.7 Statistical analysis

Continuous variables were summarized as medians with interquartile ranges (IQRs), and categorical variables as frequencies and percentages. Univariate logistic regression analyses were performed separately in the adalimumab and etanercept cohorts to evaluate the association between age and the reporting of male sexual dysfunction. Age was categorized as 18–64.9 years and ≥65 years, with the 18–64.9-year group serving as the reference category. Body weight was excluded from the regression analysis because weight data were missing for a substantial proportion of reports and patient height was unavailable in FAERS, precluding calculation of body mass index. Odds ratios (ORs) with 95% confidence intervals (CIs) were reported. Statistical significance was defined as a two-sided P value <0.05. All analyses were conducted using R (RStudio, Posit Software).

## 3 Results

### 3.1 Overview of adverse drug reaction reports from VigiAccess

Table 1 summarizes adverse drug reaction (ADR) reports for the five TNF-α inhibitors retrieved from the VigiAccess database (updated 7 June 2025), with a focus on sexual dysfunction–related events. The total number of ADR reports was highest for adalimumab (780,051) and lowest for golimumab (58,513). Male patients accounted for approximately 21%–36% of all reports.

**TABLE 1:** Sexual dysfunction-related data from VIGIACCESS public page (data is up to 7 June 2025).

Category	Adalimumab	Infliximab	Etanercept	Golimumab	Certolizumab pegol
Number of ADR reports	780,051	208,489	594,272	58,513	87,624
Male	251,227 (32%)	74,763 (36%)	145,047 (24%)	15,996 (27%)	18,731 (21%)
Reproductive system and breast disorders	9,310 (1%)	1,845 (0.88%)	4,340 (0.73%)	392 (0%)	821 (0.94%)
Erectile dysfunction	271 (2.91%)	48 (2.6%)	121 (2.79%)	15 (3.83%)	17 (2.07%)
Sexual dysfunction*	45 (0.4%)	6 (0.32%)	35 (0.81%)	3 (0.77%)	2 (0.24%)
Ejaculation disorder	9 (0%)	—	—	—	—
Psychiatric disorders	34,557 (4.43%)	5,474 (2.63%)	20,523 (3.45%)	1,628 (2.78%)	3,000 (3.42%)
Libido decreased*	95 (0.27%)	18 (0.33%)	64 (0.31%)	5 (0.31%)	8 (0.27%)
Loss of libido*	44 (0.13%)	6 (0.11%)	12 (0%)	3 (0.18%)	5 (0.17%)

* The gender of the individual could not be determined from the report.

Reports categorized under “Reproductive system and breast disorders” represented approximately 1% of total ADR reports across the five agents. Among these, erectile dysfunction was the most frequently reported event, accounting for approximately 2%–4% of reports within this category. Other sexual dysfunction–related events, including sexual dysfunction, decreased libido, and loss of libido, were infrequently reported; for some of these events, sex-specific information was not available. Notably, 271 erectile dysfunction reports were recorded for adalimumab and 121 for etanercept. Because detailed case-level data were not available in VigiAccess, further analyses were conducted using the FAERS database.

Psychiatric disorders accounted for approximately 2.6%–4.4% of total ADR reports across the five agents. Within this category, libido-related terms were infrequently reported, generally accounting for less than 0.5% of psychiatric disorder reports. Overall, sexual dysfunction–related ADR reports were uncommon across the five TNF-α inhibitors.

### 3.2 FAERS database analysis: TNF-α inhibitors vs. controls

Table 2 summarizes the characteristics of male patients reporting sexual dysfunction–related preferred terms (PTs) in the FAERS database. Anti-TNF agents were compared with the positive and negative control drugs described in the Methods section (finasteride and omeprazole, respectively).

**TABLE 2:** Patient characteristics in the FAERS database: TNF-alpha inhibitors vs. controls.

Characteristic	Adalimumab	Infliximab	Etanercept	Golimumab	Certolizumab pegol	Finasteride	Omeprazole
	(N = 149)	(N = 23)	(N = 108)	(N = 10)	(N = 14)	(N = 2,415)	(N = 131)
Age (years)							
18–64.9	136 (91.3%)	23 (100%)	97 (89.8%)	9 (90.0%)	12 (85.7%)	2,285 (94.6%)	109 (83.2%)
≥65	13 (8.7%)	0 (0%)	11 (10.2%)	1 (10.0%)	2 (14.3%)	130 (5.4%)	22 (16.8%)
Weight (kg)							
<50	1 (0.7%)	0 (0%)	0 (0%)	0 (0%)	0 (0%)	3 (0.1%)	0 (0%)
50–100	54 (36.2%)	9 (39.1%)	17 (15.7%)	3 (30.0%)	5 (35.7%)	1,639 (67.9%)	61 (46.6%)
>100	8 (5.4%)	3 (13.0%)	3 (2.8%)	0 (0%)	0 (0%)	120 (5.0%)	6 (4.6%)
missing	86 (57.7%)	11 (47.8%)	88 (81.5%)	7 (70.0%)	9 (64.3%)	653 (27.0%)	64 (48.9%)
Outcome							
Congenital anomaly	0 (0%)	0 (0%)	0 (0%)	0 (0%)	0 (0%)	3 (0.1%)	0 (0%)
Death	1 (0.7%)	0 (0%)	0 (0%)	0 (0%)	0 (0%)	47 (1.9%)	0 (0%)
Disability	4 (2.7%)	1 (4.3%)	2 (1.9%)	3 (30.0%)	2 (14.3%)	687 (28.4%)	10 (7.6%)
Hospitalization	26 (17.4%)	1 (4.3%)	13 (12.0%)	0 (0%)	0 (0%)	141 (5.8%)	10 (7.6%)
Life-Threatening	2 (1.3%)	0 (0%)	2 (1.9%)	0 (0%)	0 (0%)	163 (6.7%)	2 (1.5%)
Other	116 (77.9%)	21 (91.3%)	91 (84.3%)	7 (70.0%)	12 (85.7%)	1,374 (56.9%)	109 (83.2%)
Reporter type							
Consumer	78 (52.3%)	7 (30.4%)	32 (29.6%)	3 (30.0%)	3 (21.4%)	1705 (70.6%)	43 (32.8%)
Health professional	1 (0.7%)	4 (17.4%)	1 (0.9%)	1 (10.0%)	2 (14.3%)	55 (2.3%)	23 (17.6%)
Pharmacist	10 (6.7%)	0 (0%)	3 (2.8%)	2 (20.0%)	1 (7.1%)	33 (1.4%)	2 (1.5%)
Physician	44 (29.5%)	10 (43.5%)	53 (49.1%)	4 (40.0%)	4 (28.6%)	230 (9.5%)	23 (17.6%)
Missing	16 (10.7%)	2 (8.7%)	19 (17.6%)	0 (0%)	4 (28.6%)	392 (16.2%)	40 (30.5%)
Reporter country (top 3)							
​	US, 63 (42.3%)	US, 5 (21.7%)	US (80.74%)	FR, 3 (30.0%)	US (7.50%)	US, 1078 (44.6%)	US (38.29%)
​	CNS (46.31%)	CA, 9 (39.1%)	BR, 6 (5.5%)	CA, 2 (20.0%)	CA, 2 (14.3%)	UK, 594 (24.6%)	UK, 34 (25.9%)
​	UK, 11 (7.4%)	JP, 1 (4.3%)	CA, 3 (2.8%)	US, 2 (20.0%)	ES, 2 (14.3%)	IT, 158 (6.5%)	FR, 15 (11.5%)

BR, Brazil; CA, Canada; CNS, Country not specified; ES, Spain; FR, France; JP, Japan; IT, Italy; UK, United Kingdom; US, United States.

#### 3.2.1 Demographics and exposure

Across the study groups, most reports with available age information involved adults aged 18–64.9 years, whereas patients aged ≥65 years represented a smaller proportion. Body weight data were frequently missing, and among reports with available weight information, most patients weighed between 50 and 100 kg.

#### 3.2.2 Clinical outcomes

Among the TNF-α inhibitors, adalimumab had the largest number of male sexual dysfunction cases (n = 149), followed by etanercept (n = 108) and infliximab (n = 23). Death was infrequently reported, while hospitalization was reported in approximately 12%–17% of adalimumab and etanercept cases. Golimumab had a relatively high proportion of reports classified as disability (30.0%), although the number of cases was small (n = 10).

#### 3.2.3 Reporter characteristics

Reporter characteristics varied across the study groups, with healthcare professionals and consumers accounting for substantial proportions of reports. Most reports originated from the United States, although the geographic distribution varied across drug groups (Table 2).

### 3.3 Results of disproportionality analysis

Table 3 presents the overall disproportionality analysis of male sexual dysfunction Preferred Terms (PTs) reported with TNF-α inhibitors, with finasteride and omeprazole serving as the positive and negative controls, respectively.

**TABLE 3:** Overall disproportionality analysis of male sexual dysfunction reporting for TNF-α inhibitors and control drugs.

Drug	ROR (95% CI)	PRR (95% CI)	EBGM (EBGM05)	IC (IC025)
Adalimumab	0.2 (0.17–0.23)	0.20 (0.17–0.23)	0.2 (0.18)	−2.3 (−2.53)
Infliximab	0.11 (0.07–0.16)	0.11 (0.07–0.16)	0.11 (0.08)	−3.23 (−3.81)
Etanercept	0.21 (0.18–0.25)	0.21 (0.18–0.25)	0.21 (0.18)	−2.22 (−2.48)
Golimumab	0.22 (0.12–0.4)	0.22 (0.12–0.40)	0.22 (0.13)	−2.19 (−3.02)
Certolizumab pegol	0.25 (0.15–0.41)	0.25 (0.15–0.41)	0.25 (0.16)	−2 (−2.72)
Finasteride	76.48 (73.89–79.16)	65.99 (64.02–68.02)	56.64 (55.03)	5.82 (5.77)
Omeprazole	0.86 (0.73–1.01)	0.86 (0.74–1.01)	0.86 (0.76)	−0.21 (−0.45)

Table 2 reports deduplicated case counts, whereas the disproportionality analyses in Table 3 are based on target PT, report counts. A single deduplicated case may contain more than one target male sexual dysfunction PT, and may therefore contribute more than one PT-level record.

For all TNF-α inhibitors, ROR, PRR, and EBGM estimates for male sexual dysfunction were below 1 (ROR range: 0.11–0.25), while IC values were negative (range: −3.23 to −2.00). None of the TNF-α inhibitors met the predefined criteria for a positive overall disproportionality signal. These findings indicate that male sexual dysfunction was not disproportionately reported for adalimumab, infliximab, etanercept, golimumab, or certolizumab pegol at the overall database level.

In contrast, finasteride, the positive control, showed a strong disproportionality signal (ROR = 76.48; IC = 5.82), consistent with its established association with male sexual dysfunction. Omeprazole, the negative control, showed no positive disproportionality signal (ROR = 0.86; IC = −0.21).

### 3.4 Confounder analysis

#### 3.4.1 Concomitant medication use

Table 4 summarizes the distribution of concomitant medications with potential direct or indirect effects on male sexual function among the three study groups. In the omeprazole group, 74.8% of patients were using concomitant medications with potential direct or indirect effects on sexual function, a higher proportion than that observed in the adalimumab (41.6%) and etanercept (35.2%) groups. This group also exhibited the highest total number of concomitant drug classes (365), compared with 361 for adalimumab and 153 for etanercept.

**TABLE 4:** Concomitant medication use and the impact on sexual function in patients.

Drug	Total drug classes	Direct-acting (%)	Indirect-acting (%)	Both (%)	Total affected (%)
Adalimumab (n = 149)	361	51 (34.2%)	25 (16.8%)	14 (9.40%)	62 (41.6%)
Etanercept (n = 108)	153	37 (34.3%)	8 (7.41%)	7 (6.48%)	38 (35.2%)
Omeprazole (n = 131)	365	72 (55.0%)	47 (35.9%)	21 (16.0%)	98 (74.8%)

The high prevalence of concomitant medications with potential effects on sexual function and the substantial polypharmacy observed in the omeprazole group indicate a considerable potential for confounding by concomitant medications and underlying comorbidities when interpreting sexual dysfunction reports in this group.

#### 3.4.2 Association of age with sexual dysfunction

Univariate logistic regression showed that patients aged ≥65 years had significantly lower odds of reporting male sexual dysfunction than those aged 18–64.9 years in both cohorts: adalimumab (OR = 0.37, 95% CI: 0.18–0.73; P = 0.004) and etanercept (OR = 0.35, 95% CI: 0.19–0.66; P = 0.001) (Supplementary Tables S5, S6, respectively).

In both the adalimumab and etanercept cohorts, male sexual dysfunction reports were most frequently observed among patients aged 50–59 years, followed by those aged 40–49 years and 60–69 years (Figure 1).

**FIGURE 1:**
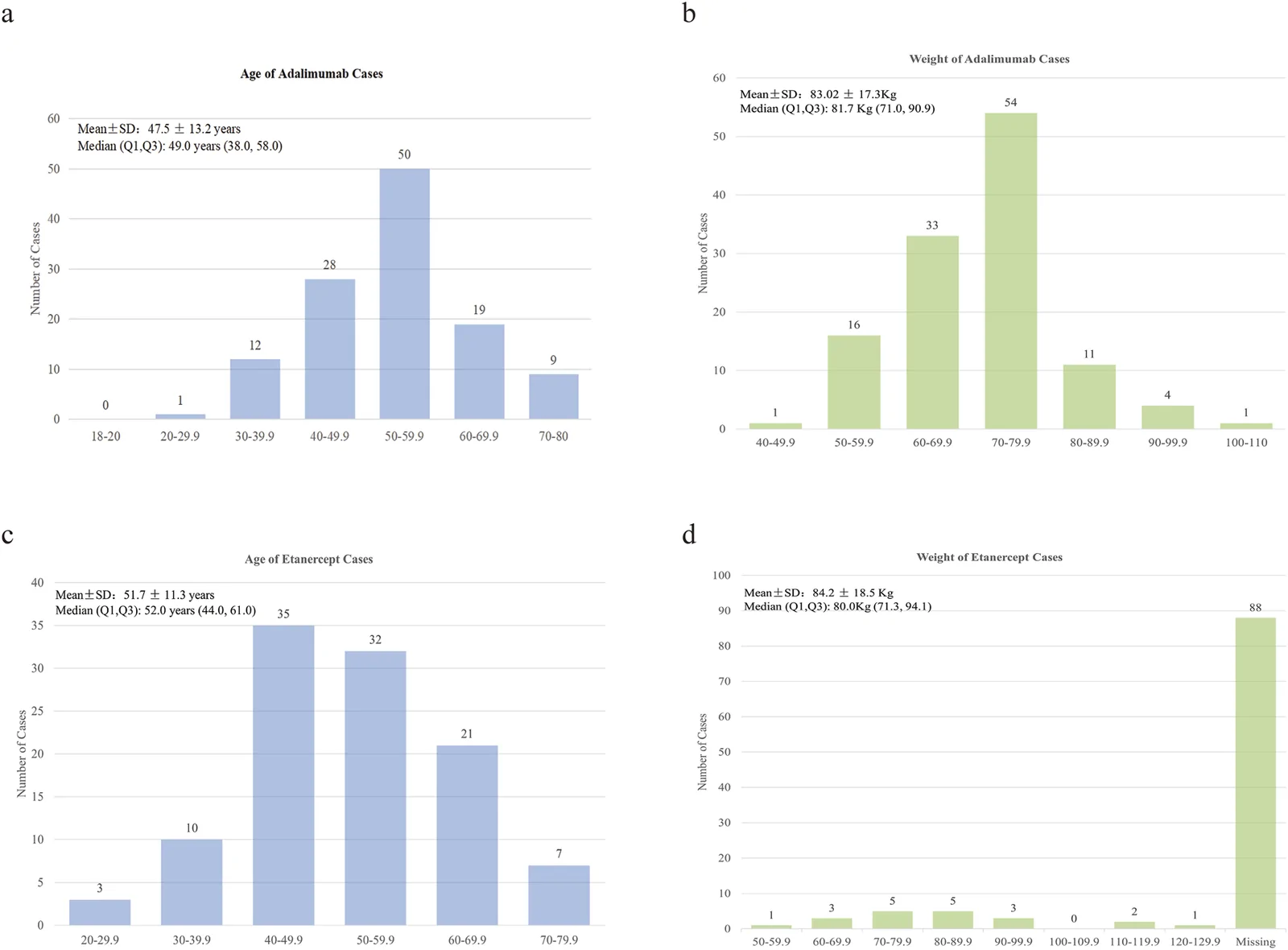
**(A)** age distribution of adalimumab cases; **(B)** body weight distribution of adalimumab cases; **(C)** age distribution of etanercept cases; and **(D)** body weight distribution of etanercept cases.

#### 3.4.3 Indications for drug use

Table 5 summarizes the most frequently reported indications among the adalimumab and etanercept cases. Psoriasis (32.2%) and Crohn’s disease (22.2%) were the most frequently reported indications for adalimumab, whereas rheumatoid arthritis (34.3%) and psoriasis (28.7%) were the most frequently reported indications for etanercept.

**TABLE 5:** Top 5 Indications for use with adalimumab and etanercept.

Adalimumab (n = 149)			Etanercept (n = 108)		
Indication	n	%	Indication	n	%
Psoriasis (including psoriatic arthropathy)	48	32.2%	Rheumatoid arthritis	37	34.3%
Crohn’s disease	33	22.2%	Psoriasis (including psoriatic arthropathy)	31	28.7%
Rheumatoid arthritis	24	16.1%	Ankylosing spondylitis	21	19.4%
Ankylosing spondylitis	19	12.8%	Unknown indication (combined)	16	14.8%
Product used for unknown indication	8	5.4%	Others	13	12%
Others	17	11.4%	​	​	​
Total	149	100%	Total	108	100%

### 3.5 Indication-specific heterogeneity in drug safety signals

#### 3.5.1 Indication-specific disproportionality analysis

Stratified ROR analysis demonstrated indication-specific differences in the reporting of male sexual dysfunction (Table 6). Statistically significant ROR-based disproportionality signals were observed in the ankylosing spondylitis (AS) cohort for both adalimumab and etanercept, whereas no statistically significant ROR signals were identified in the remaining indications.

**TABLE 6:** Indication-specific disproportionality analysis and inter-drug comparison of male sexual dysfunction reporting.

Adalimumab indication	Adalimumab ROR (95% CI)	Etanercept indication	Etanercept ROR (95% CI)	Common indication	ROR ratio (Adalimumab/Etanercept) (95% CI)	Statistically significant difference
Psoriasis	1.21 (0.86–1.70)	Rheumatoid Arthritis	0.86 (0.58–1.28)	Psoriasis	1.64 (0.96–2.79)	No
Crohn’s disease	0.78 (0.53–1.15)	Psoriasis	0.74 (0.49–1.12)	Ankylosing spondylitis	0.81 (0.41–1.61)	No
Rheumatoid arthritis	0.82 (0.53–1.27)	Ankylosing Spondylitis	2.21 (1.37–3.55)*	Rheumatoid arthritis	0.96 (0.53–1.73)	No
Ankylosing spondylitis	1.80 (1.11–2.92)*	Product used For unknown indication	1.13 (0.65–1.94)	—	—	—

* A positive signal was defined as a ≥3 and a 95% CI, lower bound >1.0.

The comparative odds ratio (ROR ratio) analysis showed no statistically significant difference between adalimumab and etanercept across the common indications evaluated. The ROR ratio was 1.64 (95% CI: 0.96–2.79) in psoriasis and 0.81 (95% CI: 0.41–1.61) in ankylosing spondylitis, with all 95% confidence intervals including 1.0 (Table 6). The corresponding indication-stratified results based on PRR, BCPNN, and MGPS are presented in Supplementary Table S3.

#### 3.5.2 Stratified results of sexual dysfunction reporting

Figure 2 summarizes the distribution of reported male sexual dysfunction Preferred Terms (PTs) for adalimumab and etanercept. Erectile dysfunction (ED) was the most frequently reported PT for both drugs, followed by libido-related terms, including Libido Decreased and Loss of Libido.

**FIGURE 2:**
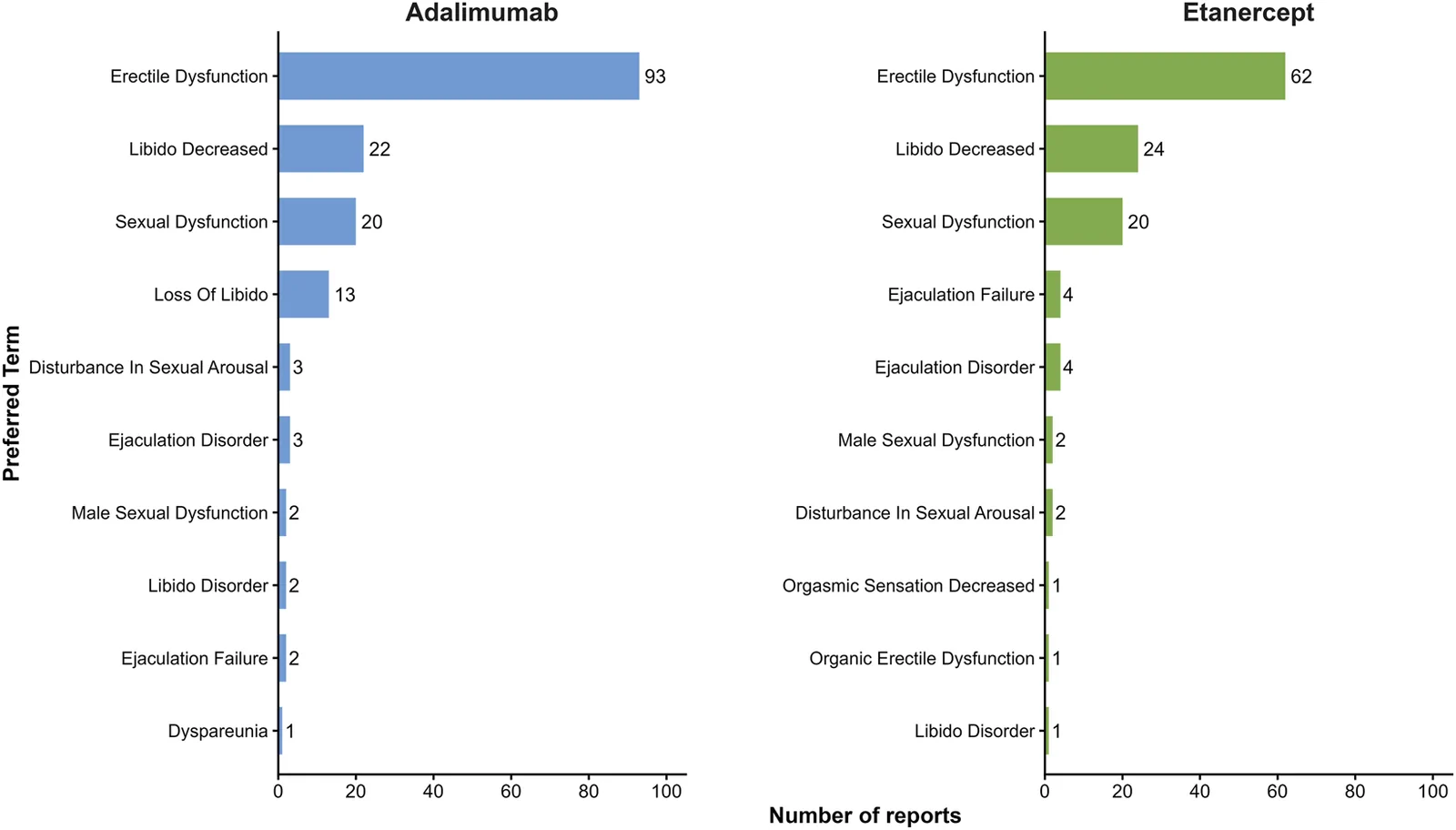
Distribution of reported male sexual dysfunction preferred terms for adalimumab and etanercept in the FAERS database. Bars represent the number of reports containing each preferred term. A single report may contain more than one preferred term; therefore, PT counts are not mutually exclusive case counts.

To further characterize PT-specific reporting patterns, stratified ROR analyses were performed within the psoriasis (Ps) and ankylosing spondylitis (AS) cohorts. The results are summarized in Table 7, with the corresponding PRR, BCPNN, and MGPS results presented in Supplementary Table S4.

**TABLE 7:** Preferred term-specific disproportionality analysis of male sexual dysfunction reporting within psoriasis and ankylosing spondylitis strata.

Drug	Indication	Preferred term (PT)	a	ROR (95% CI)	Statistical interpretation
Adalimumab	Psoriasis	Erectile dysfunction	37	1.00 (0.70–1.44)	No
		Libido decreased/Loss	7	0.67 (0.30–1.50)	No
		Sexual dysfunction	5	0.37 (0.15–0.93)	Significantly lower reporting
		Male sexual dysfunction	1	1.77 (0.16–19.55)	No
		Dyspareunia	1	Not estimable	Not estimable (zero comparator count)
		Ejaculation disorder	1	1.18 (0.12–11.36)	No
		Disturbance in sexual arousal	1	3.55 (0.22–56.68)	No
Etanercept	Psoriasis	Erectile dysfunction	16	0.41 (0.25–0.69)	Significantly lower reporting
		Sexual dysfunction	7	0.59 (0.27–1.32)	No
		Libido decreased/Loss	8	1.08 (0.49–2.36)	No
		Ejaculation disorder	3	2.93 (0.66–13.10)	No
		Disturbance in sexual arousal	1	3.91 (0.24–62.51)	No
		Male sexual dysfunction	1	1.95 (0.18–21.56)	No
Adalimumab	Ankylosing spondylitis	Erectile dysfunction	10	0.62 (0.31–1.22)	No
		Libido decreased/Loss	8	2.17 (0.85–5.49)	No
		Sexual dysfunction	5	0.90 (0.33–2.48)	No
		Ejaculation disorder	1	1.35 (0.12–14.93)	No
Etanercept	Ankylosing spondylitis	Erectile dysfunction	18	1.47 (0.83–2.59)	No
		Sexual dysfunction	5	0.98 (0.36–2.69)	No
		Libido decreased/Loss	5	1.83 (0.60–5.61)	No

For each drug-PT, pair, a denotes the number of reports containing the specified PT, for the drug of interest within the corresponding indication stratum; the complete underlying 2 × 2 cell counts (a, b, c, and d) for all analyses are provided in Supplementary Appendix C. RORs, were calculated against all other drugs in FAERS, within the respective indication strata. A positive signal was defined as a ≥3 and a 95% CI, lower bound >1.0. Rows containing a 0 cell were not estimable using the prespecified uncorrected ROR, formula.

Within the AS stratum, no statistically significant positive ROR-based PT-specific signal was identified for either adalimumab or etanercept. For etanercept, the RORs were 1.47 (95% CI: 0.83–2.59) for erectile dysfunction, 0.98 (95% CI: 0.36–2.69) for sexual dysfunction, and 1.83 (95% CI: 0.60–5.61) for libido decreased/loss. Similarly, no statistically significant positive ROR-based PT-specific signal was observed for adalimumab.

Within the Ps stratum, most PT-specific estimates were not statistically significant. Adalimumab showed significantly lower reporting for sexual dysfunction (ROR = 0.37, 95% CI: 0.15–0.93), whereas etanercept showed significantly lower reporting for erectile dysfunction (ROR = 0.41, 95% CI: 0.25–0.69). No positive ROR-based PT-specific signal was detected for either drug.

Overall, although ROR-based indication-level disproportionality signals were observed for both adalimumab and etanercept in AS, these signals were not accompanied by a statistically significant increase in the reporting of any individual sexual dysfunction PT examined.

#### 3.5.3 Adverse event onset profile

Table 8 and Figure 3 summarize the Weibull analysis of time-to-onset (TTO) for male sexual dysfunction reports associated with adalimumab and etanercept, including analyses of overall male sexual dysfunction and erectile dysfunction (ED), the most frequently reported PT.

**TABLE 8:** Weibull analysis of time-to-onset.

Drug/ADR*	Shape parameter (β) (95% CI)	Scale parameter (α) (days) (95% CI)	Time-to-onset quantiles (days) (0%, 25%, 50%, 75%, 100%)	Reporting-time pattern
Adalimumab (overall, n = 38)	0.68 (0.51–0.86)	276.54 (140.89–412.18)	1, 40.8, 165.0, 452.3, 1,393	Earlier reporting pattern
Etanercept (overall, n = 17)	0.63 (0.40–0.86)	299.80 (60.65–538.96)	2, 59, 124, 409, 2,385	Earlier reporting pattern
*Adalimumab (ED, n = 28)	0.71 (0.50–0.93)	322.84 (146.55–499.12)	1, 70.8, 196.5, 505.5, 1,393	Earlier reporting pattern
*Etanercept (ED, n = 13)	0.61 (0.35–0.86)	322.50 (18.42–626.58)	2, 59, 124, 409, 2,385	Earlier reporting pattern

* ED, Erectile dysfunction.

β < 1 indicates that reported events are more concentrated earlier after treatment initiation; β = 1 indicates an approximately constant reporting pattern; and β > 1 indicates a later reporting pattern.

**FIGURE 3:**
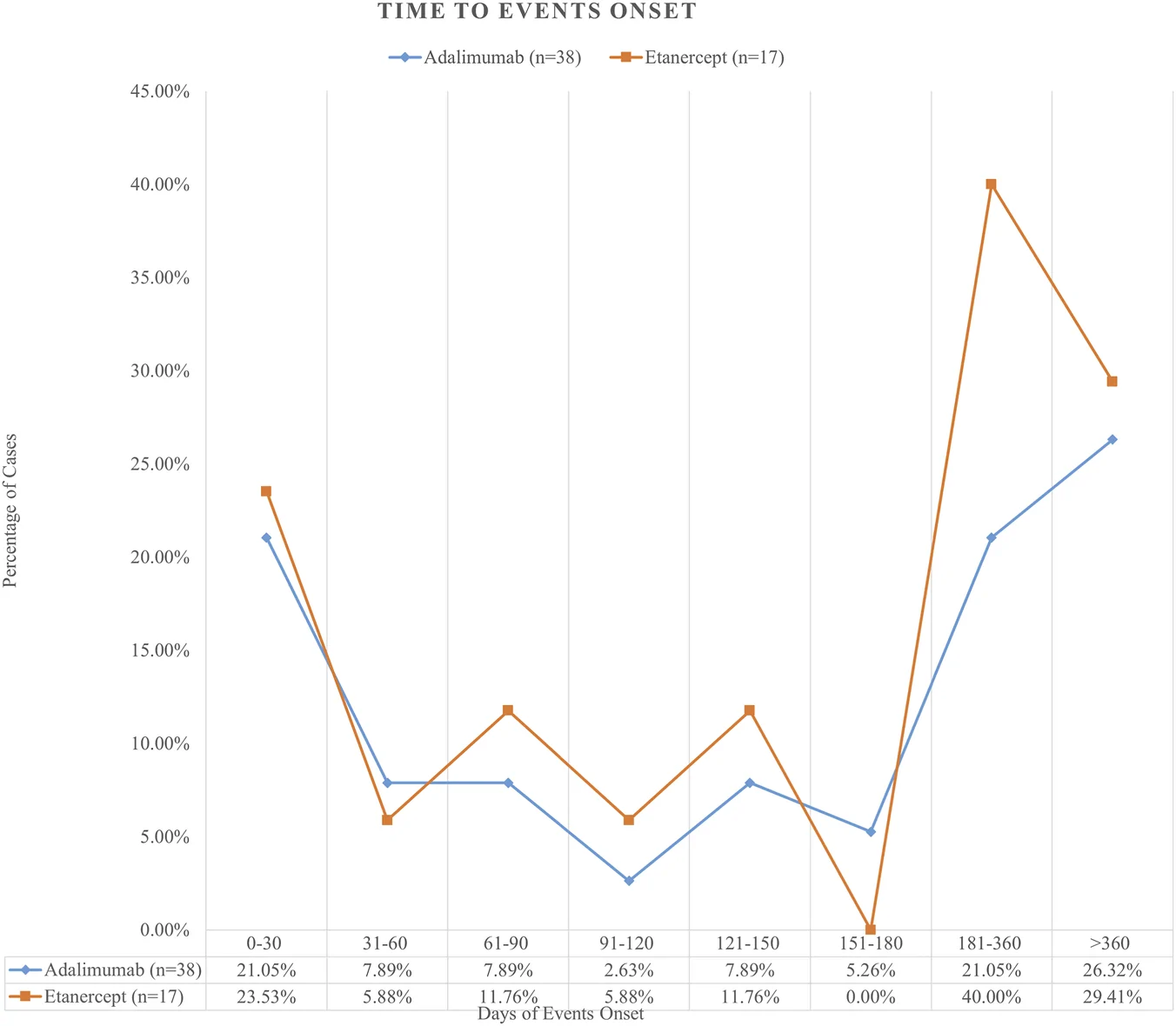
Time-to-onset distribution of male sexual dysfunction reports. The analysis included reports with usable treatment-start and event-onset dates (adalimumab, n = 38; etanercept, n = 17).

For adalimumab, the overall TTO analysis (n = 38) yielded a shape parameter (β) of 0.68 (95% CI: 0.51–0.86), with a median TTO of approximately 165 days. In the ED-specific analysis (n = 28), the β value was 0.71 (95% CI: 0.50–0.93), with a median TTO of approximately 197 days.

For etanercept, the overall TTO analysis yielded a shape parameter (β) of 0.63 (95% CI: 0.40–0.86), with a median TTO of 124 days. In the ED-specific analysis (n = 13), the β value was 0.61 (95% CI: 0.35–0.86), with a median TTO of 124 days.

All β values were less than 1, indicating that reported events were more concentrated earlier after treatment initiation. These findings describe an early reporting-time pattern rather than the temporal distribution of clinical risk. The interquartile ranges of TTO were approximately 41–452 days for adalimumab and 59–409 days for etanercept, indicating substantial overlap in the observed reporting-time ranges between the two agents.

## 4 Discussion

Our findings revealed indication-related heterogeneity in the reporting of male sexual dysfunction among TNF-α inhibitors. In the overall FAERS analysis, none of the evaluated TNF-α inhibitors showed a positive disproportionality signal for male sexual dysfunction. However, indication-stratified analyses identified statistically significant ROR-based signals for both adalimumab and etanercept in ankylosing spondylitis (AS), whereas no statistically significant signals were observed in the other major indications evaluated. Importantly, direct comparison between adalimumab and etanercept showed no statistically significant difference in reporting disproportionality across the common indications. Furthermore, PT-specific analyses within AS identified no statistically significant positive ROR signal for either agent. Taken together, these findings suggest that MSD reporting may vary according to the underlying clinical indication rather than representing a uniform class-wide or clearly drug-specific pattern.

Although the overall indication-stratified analysis did not demonstrate a statistically significant difference between adalimumab and etanercept in AS, differences in their molecular structures and target-binding profiles may nevertheless contribute to differences in observed reporting patterns. Etanercept is a p75 TNF receptor–IgG1 fusion protein that binds both TNF-α and lymphotoxin-α (LT-α), whereas adalimumab is a fully human IgG1 monoclonal antibody directed against TNF-α (Lim et al., 2018). Importantly, AS itself has been associated with erectile dysfunction (Zhang et al., 2023) and endothelial dysfunction (Łosińska et al., 2019), indicating that the underlying disease may contribute to the observed reporting patterns. However, the potential relevance of these biological differences to male sexual dysfunction remains speculative and cannot be established from pharmacovigilance data alone.

The TTO analysis provided descriptive information on reporting-time patterns among the limited subset of reports with usable treatment-start and event-onset dates. The median TTO for overall male sexual dysfunction was 165 days for adalimumab and 124 days for etanercept. Weibull shape parameters (β < 1) suggested that reported events were relatively more concentrated earlier after treatment initiation. However, because TTO information was available for only a limited subset of reports and FAERS does not provide a defined exposed population, these findings should be interpreted solely as reporting-time patterns and not as evidence that the clinical risk of sexual dysfunction is highest during a particular treatment period.

Metabolic and cardiovascular comorbidities may also confound the relationship between TNF-α inhibitor use and male sexual dysfunction. Obesity (McNabney et al., 2023), diabetes (Dilixiati et al., 2024), and hypertension (Wang et al., 2018) are well-established contributors to erectile dysfunction and may therefore confound the reporting of male sexual dysfunction. Body weight was not included in the regression analysis because weight data were missing for a substantial proportion of reports and patient height was unavailable in FAERS, precluding calculation of body mass index. In addition, antidiabetic medication use was used only as a proxy for diabetes and therefore could not fully capture the contribution of underlying metabolic disease. These limitations highlight the difficulty of distinguishing drug-related effects from disease- and comorbidity-related influences in spontaneous reporting data.

Age may influence both the occurrence and reporting of male sexual dysfunction. Although erectile dysfunction becomes more prevalent with advancing age in population-based studies (Feldman et al., 1994), our analysis showed that reports were concentrated among middle-aged patients. In the univariate analyses, patients aged ≥65 years had lower odds of reporting male sexual dysfunction than those aged 18–64.9 years for both adalimumab and etanercept. This apparent discrepancy between the expected age-related prevalence of erectile dysfunction and the reporting pattern observed in FAERS may reflect differences in healthcare-seeking behavior, treatment exposure, or reporting practices across age groups. Therefore, the lower reporting odds observed in older patients should not be interpreted as evidence of a lower underlying risk of sexual dysfunction.

Our indication-stratified analyses further highlighted the importance of the underlying disease context. Statistically significant indication-level disproportionality signals were observed for both adalimumab and etanercept in the AS cohort, whereas no statistically significant signals were identified in the other major indications evaluated. However, these indication-level findings were not accompanied by statistically significant positive ROR-based signals for individual sexual dysfunction PTs within the AS stratum. This discrepancy may partly reflect the broader event definition and larger number of reports contributing to the composite MSD outcome compared with individual PTs, for which report counts were substantially smaller. Accordingly, the indication-level findings should not be interpreted as evidence of a specific sexual dysfunction phenotype or a drug-specific effect. Rather, they support the possibility that disease-related factors, patient characteristics, treatment patterns, and residual confounding collectively contribute to the observed reporting heterogeneity.

Potential confounding and reporting biases must also be considered. Many patients used concomitant medications known to affect sexual function—such as antidepressants, β-blockers, and lipid-lowering agents (Corona et al., 2025; Adeeb et al., 2025; Montejo et al., 2019). Because FAERS lacks complete diagnostic information, antidiabetic drug use was employed as a proxy for diabetes. The high prevalence of these medications suggests that polypharmacy and comorbidity may have contributed to the observed reporting patterns. In addition, body weight information was missing for a substantial proportion of reports, and patient height was unavailable in FAERS, precluding calculation of BMI and direct assessment of obesity as a potential confounder.

The control drug analysis further illustrates the importance of considering background reporting and residual confounding. Although spontaneous reports of male sexual dysfunction were identified for omeprazole, no disproportionality signal was detected (ROR = 0.86; IC = −0.21). Given the high prevalence of concomitant medications known to affect sexual function and the inherent reporting biases of spontaneous reporting systems, these reports may reflect background reporting and residual confounding rather than a specific pharmacological association. Therefore, the absolute number of spontaneous reports should not be interpreted as evidence of a drug-related association without considering disproportionality measures.

Several inherent limitations of spontaneous reporting systems must be acknowledged, including underreporting, potential residual duplication, and variable data completeness, which preclude causal inference. Furthermore, because FAERS does not provide a defined exposed population or incidence denominator, disproportionality measures reflect relative reporting rather than absolute or comparative clinical risk (Fusaroli et al., 2024). In addition, disproportionality estimates may vary across analytical methods, particularly when based on small numbers of reports. The use of multiple disproportionality algorithms and indication-specific analyses therefore provides complementary perspectives rather than definitive confirmation of a safety signal. Accordingly, the present findings should be regarded as hypothesis-generating and require validation in prospective studies.

From a clinical perspective, sexual dysfunction can substantially affect quality of life and treatment adherence, even though such reports were relatively uncommon in the present pharmacovigilance analysis. In patients with inflammatory diseases such as AS, assessment of sexual health may be considered as part of routine clinical evaluation, particularly because the underlying disease itself can adversely affect sexual function (Zhang et al., 2023). If new-onset sexual dysfunction is reported during TNF-α inhibitor therapy, clinicians should consider both drug-related and non-drug-related factors, including disease activity, comorbidities, concomitant medications, and psychosocial factors. Importantly, the present findings do not support preferential selection of adalimumab or etanercept on the basis of male sexual dysfunction reporting.

In conclusion, this real-world pharmacovigilance study identified indication-related heterogeneity in the reporting of male sexual dysfunction among reports involving TNF-α inhibitors. Although no overall positive disproportionality signal was detected, indication-level signals were observed for both adalimumab and etanercept in ankylosing spondylitis. However, direct comparison between the two agents showed no statistically significant difference, and no positive PT-specific signals were identified within the AS stratum. These findings suggest that the underlying disease context and residual confounding should be carefully considered when interpreting spontaneous reporting signals. Further prospective studies are needed to determine whether these reporting patterns reflect true treatment-related effects.

## Data Availability

The datasets presented in this study can be found in online repositories. The names of the repository/repositories and accession number(s) can be found below: https://fis.fda.gov/extensions/FPD-QDE-FAERS/FPD-QDE-FAERS.html; https://www.vigiaccess.org/.
