# Supplementary material for: Male sexual dysfunction associated with TNF-α inhibitors: an indication-stratified global pharmacovigilance analysis
Source: Front Pharmacol. 2026 Sep 14;17:1915246. doi: 10.3389/fphar.2026.1915246 (PMC13617334; doi:10.3389/fphar.2026.1915246)
Supplement: Supplementary file 1 [file Supplementaryfile1.docx]

**Appendix A. Analytical Framework of the Present Study**

This appendix summarizes the analytical framework used in the manuscript, including the source population, statistical unit, event definition, denominator/comparator, primary output, and key notes for each analysis. Its purpose is to facilitate reproducibility and reconciliation of the main tables and figures. one deduplicated FAERS case may contribute more than one target PT record if multiple predefined MSD PTs are reported.

| Analysis | Source population | Statistical unit | Case / event definition | Denominator & comparator | Primary output | Key notes |
| --- | --- | --- | --- | --- | --- | --- |
| Overall disproportionality | Deduplicated male FAERS reports with the drug coded as Primary Suspect | drug–PT report record | Reports of individual target MSD PTs; one deduplicated case may contribute more than one target PT record | All other adverse events in the predefined comparison background | Table 3 (ROR, PRR, EBGM, BCPNN) | Overall drug-level disproportionality analysis based on target PT report counts |
|  |  |  |  |  |  |  |
| Demographic characteristics | Deduplicated target-case cohort | Case | Each deduplicated case counted once | No comparator (descriptive) | Table 2; Figure 1 | Baseline characteristics only. |
| Concomitant medication | Same target-case cohort as Table 2 | Case | Each deduplicated case counted once | Within-drug descriptive analysis; no external comparator | Table 4 | One case may contain multiple concomitant drugs. |
|  |  |  |  |  |  |  |
| Indication distribution | Same target-case cohort as Table 2 | Case | Each deduplicated case assigned to the recorded indication(s) | Total target cases for each drug | Table 5 | Descriptive analysis; indication counts may not be mutually exclusive when multiple indications are recorded for a case. |
|  |  |  |  |  |  |  |
| Indication-specific disproportionality | FAERS reports restricted to the corresponding indication stratum | Report | Reports containing ≥1 target MSD PT within the corresponding indication stratum | All other adverse-event reports within the same indication stratum and predefined comparison background | Table 6; Supplementary Table 3 | Indication-specific disproportionality analysis |
| PT-specific disproportionality | FAERS reports restricted by drug and indication | Preferred Term (PT) | Reports containing an individual PT | Same PT in the predefined comparison background | Table 7; Figure 2; Supplementary Table 4 | A single case may contribute to multiple PTs; therefore, PT counts may exceed the number of deduplicated cases. |
|  |  |  |  |  |  |  |
| Time-to-onset (TTO) | Cases with valid treatment-start and event-onset dates | Case | Each case contributes one valid TTO value | No comparator | Figure 3 | Describes reporting-time patterns rather than clinical risk. |
| Logistic regression | Deduplicated study cohorts | Case | Target MSD event present versus absent | Age-group comparison: 18–64.9 vs ≥65 years | Supplementary Table 5–6 | Univariate analysis after exclusion of body weight. |

Abbreviations: FAERS, FDA Adverse Event Reporting System; MSD, male sexual dysfunction; PT, Preferred Term; ROR, reporting odds ratio; PRR, proportional reporting ratio; EBGM, empirical Bayes geometric mean; BCPNN, Bayesian confidence propagation neural network; TTO, time-to-onset.

**Appendix B. Figure S1 Numerical case-selection and analytical flow**

**WHO VigiAccess initial screening
TNF-α inhibitors and male sexual dysfunction reports
(Table 1)**

**↓**

**FAERS reports retrieved (2004–2025)
N = 4,678,324**

**↓ (Excluded duplicates: N = 836,588)**

**Reports after FDA-recommended deduplication
N = 3,841,736**

**↓ (Excluded not meeting criteria: N = 3,611,146)**

**Male reports involving one of the five study TNF-α inhibitors as Primary Suspect
N = 230,590**

**↓**

| **MSD event classification** | |
| --- | --- |
| **Deduplicated reports with ≥1 predefined MSD PT**  **N = 304** | **Deduplicated reports without a predefined MSD PT**  **N = 230,286** |

**↓**

| **Analytical populations required for numerical reconciliation** | | |
| --- | --- | --- |
| Indication-stratified analysis AS target-event reports: ADA a = 19; ETA a = 21 Other major indications: see Appendix C Table 2 | PT-specific analysis within AS ADA: ED 10; libido decreased/loss 8; sexual dysfunction 5; ejaculation disorder 1 ETA: ED 18; sexual dysfunction 5; libido decreased/loss 5 | Time-to-onset analysis Usable onset dates: Overall MSD: N = 55 (ADA 38; ETA 17) ED-specific: N = 41 (ADA 28; ETA 13) |

**↓**

| **Final analyses** | | | |
| --- | --- | --- | --- |
| **Overall / indication / PT disproportionality ROR · PRR · BCPNN · MGPS** | **Direct ADA vs ETA comparison + univariate age analysis** | **TTO / Weibull reporting-time patterns** | **Control drugs Finasteride / Omeprazole** |

Overall drug-specific 2×2 target-event counts (Appendix C Table 1; not unique-report partitions): adalimumab a=161; etanercept a=121; infliximab a=24; certolizumab pegol a=15; golimumab a=11.

Abbreviations: ADA, adalimumab; ETA, etanercept; AS, ankylosing spondylitis; ED, erectile dysfunction; FAERS, FDA Adverse Event Reporting System; MSD, male sexual dysfunction; PT, preferred term; PS, Primary Suspect; TTO, time to onset.

Appendix C Table 1. Underlying 2×2 contingency tables used for the overall disproportionality analyses (corresponding to Table 3).

Only the target_PT contingency tables are presented because the corresponding NO_target_PT tables represent the complementary cells of the same 2×2 contingency tables and therefore contain redundant information.

| **Drug** | **a** | **b** | **c** | **d** |
| --- | --- | --- | --- | --- |
| Adalimumab | 161 | 323352 | 29724 | 11822935 |
| Etanercept | 121 | 229384 | 29764 | 11916903 |
| Certolizumab pegol | 15 | 24448 | 29870 | 12121839 |
| Infliximab | 24 | 91994 | 29861 | 12054293 |
| Golimumab | 11 | 20400 | 29874 | 12125887 |
| Finasteride | 4301 | 26640 | 25584 | 12119647 |
| Omeprazole | 151 | 71097 | 29734 | 12075190 |

Appendix C Table 2. Underlying 2×2 contingency tables used for the indication-specific disproportionality analyses reported in Table 6 and Supplementary Table 3.

| **Drug** | **Indication** | **a** | **b** | **c** | **d** |
| --- | --- | --- | --- | --- | --- |
| Adalimumab | Psoriasis | 48 | 27,585 | 101 | 70,081 |
| Adalimumab | Crohn's disease | 33 | 26,137 | 116 | 71,529 |
| Adalimumab | Rheumatoid arthritis | 24 | 18,473 | 125 | 79,193 |
| Adalimumab | Ankylosing spondylitis | 19 | 7,320 | 130 | 90,346 |
| Adalimumab | Product used for unknown indication | 8 | 3,702 | 141 | 93,964 |
| Etanercept | Rheumatoid arthritis | 37 | 33,339 | 71 | 54,836 |
| Etanercept | Psoriasis | 31 | 31,126 | 77 | 57,049 |
| Etanercept | Ankylosing spondylitis | 21 | 8,696 | 87 | 79,479 |
| Etanercept | Drug use for unknown indication | 1 | 106 | 107 | 88,069 |
| Etanercept | Product used for unknown indication | 15 | 11,054 | 93 | 77,121 |

**Appendix C Table 3. Underlying 2×2 contingency tables used for the PT-specific disproportionality analyses reported in Table 7 and Supplementary Table 4**

| **Drug** | **Indication** | **Preferred Term (PT)** | **a** | **b** | **c** | **d** |
| --- | --- | --- | --- | --- | --- | --- |
| Adalimumab | Psoriasis | Erectile Dysfunction | 37 | 91,582 | 131 | 324,664 |
| Adalimumab | Psoriasis | Libido Decreased/Loss | 7 | 91,612 | 37 | 324,758 |
| Adalimumab | Psoriasis | Sexual Dysfunction | 5 | 91,614 | 48 | 324,747 |
| Adalimumab | Psoriasis | Male Sexual Dysfunction | 1 | 91,618 | 2 | 324,793 |
| Adalimumab | Psoriasis | Dyspareunia | 1 | 91,618 | 0 | 324,795 |
| Adalimumab | Psoriasis | Ejaculation Disorder | 1 | 91,618 | 3 | 324,792 |
| Adalimumab | Psoriasis | Disturbance in Sexual Arousal | 1 | 91,618 | 1 | 324,794 |
| Etanercept | Psoriasis | Erectile Dysfunction | 16 | 84,808 | 152 | 331,457 |
| Etanercept | Psoriasis | Sexual Dysfunction | 7 | 84,817 | 46 | 331,563 |
| Etanercept | Psoriasis | Libido Decreased/Loss | 8 | 84,816 | 29 | 331,580 |
| Etanercept | Psoriasis | Ejaculation Disorder | 3 | 84,821 | 4 | 331,605 |
| Etanercept | Psoriasis | Disturbance in Sexual Arousal | 1 | 84,823 | 1 | 331,608 |
| Etanercept | Psoriasis | Male Sexual Dysfunction | 1 | 84,823 | 2 | 331,607 |
| Adalimumab | Ankylosing Spondylitis | Erectile Dysfunction | 10 | 28,908 | 44 | 78,231 |
| Adalimumab | Ankylosing Spondylitis | Libido Decreased/Loss | 8 | 28,910 | 10 | 78,265 |
| Adalimumab | Ankylosing Spondylitis | Sexual Dysfunction | 5 | 28,913 | 15 | 78,260 |
| Adalimumab | Ankylosing Spondylitis | Ejaculation Disorder | 1 | 28,917 | 2 | 78,273 |
| Etanercept | Ankylosing Spondylitis | Erectile Dysfunction | 18 | 27,223 | 36 | 79,916 |
| Etanercept | Ankylosing Spondylitis | Sexual Dysfunction | 5 | 27,236 | 15 | 79,937 |
| Etanercept | Ankylosing Spondylitis | Libido Decreased/Loss | 5 | 27,236 | 8 | 79,944 |

**Definitions of the contingency table**

**a =** reports containing the specified Preferred Term (PT) associated with the study drug within the corresponding indication stratum.

**b =** reports of all other adverse events associated with the study drug within the corresponding indication stratum.

**c =** reports containing the same PT for all other drugs in FAERS within the corresponding indication stratum.

**d =** reports of all other adverse events for all other drugs in FAERS within the corresponding indication stratum.

**Note:** The same 2×2 contingency tables were used to calculate the ROR values reported in Table 7 and the PRR, BCPNN, and MGPS estimates reported in Supplementary Table 4.

**Appendix D Disproportionality Analysis Methods and Reproducible R Code**

*Supporting analytical documentation for ROR, PRR, BCPNN, and MGPS calculations*

**Purpose.** This document provides the exact calculation framework and executable R code used to reproduce the disproportionality analyses reported in the manuscript and Supplementary Appendix C. The same 2 × 2 contingency-table structure is used throughout, with the interpretation of a–d determined by the corresponding analytical level.

**1. General 2 × 2 Contingency Table**

|  | **Target adverse event** | **Other adverse events** | **Total** |
| --- | --- | --- | --- |
| **Drug of interest** | a | b | a + b |
| **All other drugs / comparison background** | c | d | c + d |
| **Total** | a + c | b + d | N = a + b + c + d |

Definitions: a = target-event reports for the drug of interest; b = other adverse-event reports for the drug of interest; c = target-event reports in the comparison background; d = other adverse-event reports in the comparison background; N = a + b + c + d.

**2. Reporting Odds Ratio (ROR)**

| **Formula** | ROR = (a × d) / (b × c) |
| --- | --- |
| **Standard error** | SE[ln(ROR)] = sqrt(1/a + 1/b + 1/c + 1/d) |
| **95% confidence interval** | exp{ ln(ROR) ± 1.96 × SE[ln(ROR)] } |
| **Signal criterion** | a ≥ 3 and the lower limit of the 95% CI > 1 |

**3. Proportional Reporting Ratio (PRR)**

| **Formula** | PRR = [a/(a+b)] / [c/(c+d)] |
| --- | --- |
| **Standard error** | SE[ln(PRR)] = sqrt(1/a − 1/(a+b) + 1/c − 1/(c+d)) |
| **95% confidence interval** | exp{ ln(PRR) ± 1.96 × SE[ln(PRR)] } |
| **Signal criterion** | a ≥ 3 and the lower limit of the 95% CI > 1 |

**4. Bayesian Confidence Propagation Neural Network (BCPNN)**

| **Prior parameters** | α1 = β1 = 1; α = β = 2; γ11 = 1 |
| --- | --- |
| **Auxiliary parameter** | γ = γ11 × [(N+α)(N+β)] / [(a+b+α1)(a+c+β1)] |
| **Expected information component** | E(IC) = log2{ [(a+γ11)(N+α)(N+β)] / [(N+γ)(a+b+α1)(a+c+β1)] } |
| **Variance** | V(IC) = 1/(ln2)^2 × { [N−a+γ−γ11]/[(a+γ11)(1+N+γ)] + [N−(a+b)+α−α1]/[(a+b+α1)(1+N+α)] + [N−(a+c)+β−β1]/[(a+c+β1)(1+N+β)] } |
| **Lower credibility bound** | IC025 = E(IC) − 2 × sqrt[V(IC)] |
| **Signal criterion** | a ≥ 3 and IC025 > 0 |

**5. Multi-item Gamma Poisson Shrinker (MGPS) Calculation Used in This Study**

| **EBGM** | EBGM = a × N / [(a+c)(a+b)] |
| --- | --- |
| **Lower one-sided 95% confidence limit** | EBGM05 = exp{ ln(EBGM) − 1.64 × sqrt(1/a + 1/b + 1/c + 1/d) } |
| **Signal criterion** | a > 0 and EBGM05 > 2.0 |

**6. Unified R Function**

\# ============================================================

\# Disproportionality analysis from 2 x 2 contingency tables

\# Methods: ROR, PRR, BCPNN, and MGPS

\# ============================================================

calc_signal <- function(a, b, c, d) {

N <- a + b + c + d

\# 1. Reporting Odds Ratio (ROR)

if (all(c(a, b, c, d) > 0)) {

ROR <- (a * d) / (b * c)

SE_log_ROR <- sqrt(1/a + 1/b + 1/c + 1/d)

ROR_lower <- exp(log(ROR) - 1.96 * SE_log_ROR)

ROR_upper <- exp(log(ROR) + 1.96 * SE_log_ROR)

} else {

ROR <- ROR_lower <- ROR_upper <- NA_real_

}

\# 2. Proportional Reporting Ratio (PRR)

if (a > 0 && c > 0) {

PRR <- (a / (a + b)) / (c / (c + d))

SE_log_PRR <- sqrt(1/a - 1/(a+b) + 1/c - 1/(c+d))

PRR_lower <- exp(log(PRR) - 1.96 * SE_log_PRR)

PRR_upper <- exp(log(PRR) + 1.96 * SE_log_PRR)

} else {

PRR <- PRR_lower <- PRR_upper <- NA_real_

}

\# 3. Bayesian Confidence Propagation Neural Network (BCPNN)

alpha1 <- 1

beta1 <- 1

alpha <- 2

beta <- 2

gamma11 <- 1

gamma <- gamma11 * ((N + alpha) * (N + beta)) /

((a + b + alpha1) * (a + c + beta1))

E_IC <- log2(

((a + gamma11) * (N + alpha) * (N + beta)) /

((N + gamma) * (a + b + alpha1) * (a + c + beta1))

)

V_IC <- (1 / (log(2)^2)) * (

(N - a + gamma - gamma11) /

((a + gamma11) * (1 + N + gamma)) +

(N - (a + b) + alpha - alpha1) /

((a + b + alpha1) * (1 + N + alpha)) +

(N - (a + c) + beta - beta1) /

((a + c + beta1) * (1 + N + beta))

)

IC025 <- E_IC - 2 * sqrt(V_IC)

\# 4. MGPS calculation used in this study

EBGM <- a * N / ((a + c) * (a + b))

if (all(c(a, b, c, d) > 0)) {

EBGM05 <- exp(

log(EBGM) - 1.64 * sqrt(1/a + 1/b + 1/c + 1/d)

)

} else {

EBGM05 <- NA_real_

}

\# Signal criteria

ROR_signal <- !is.na(ROR_lower) && a >= 3 && ROR_lower > 1

PRR_signal <- !is.na(PRR_lower) && a >= 3 && PRR_lower > 1

BCPNN_signal <- a >= 3 && IC025 > 0

MGPS_signal <- !is.na(EBGM05) && a > 0 && EBGM05 > 2

data.frame(

a = a, b = b, c = c, d = d,

ROR = ROR, ROR_lower = ROR_lower, ROR_upper = ROR_upper,

PRR = PRR, PRR_lower = PRR_lower, PRR_upper = PRR_upper,

E_IC = E_IC, IC025 = IC025,

EBGM = EBGM, EBGM05 = EBGM05,

ROR_signal = ROR_signal,

PRR_signal = PRR_signal,

BCPNN_signal = BCPNN_signal,

MGPS_signal = MGPS_signal

)

}

apply_signal_function <- function(dat) {

out <- do.call(

rbind,

lapply(seq_len(nrow(dat)), function(i) {

calc_signal(dat$a[i], dat$b[i], dat$c[i], dat$d[i])

})

)

cbind(dat, out[, setdiff(names(out), c("a", "b", "c", "d"))])

}

**7. Code for Supplementary Appendix C Table 1 (Overall Disproportionality; reproduces manuscript Table 3)**

appendixC_table1 <- data.frame(

Drug = c("Adalimumab", "Etanercept", "Certolizumab pegol",

"Infliximab", "Golimumab", "Finasteride", "Omeprazole"),

a = c(161, 121, 15, 24, 11, 4301, 151),

b = c(323352, 229384, 24448, 91994, 20400, 26640, 71097),

c = c(29724, 29764, 29870, 29861, 29874, 25584, 29734),

d = c(11822935, 11916903, 12121839, 12054293,

12125887, 12119647, 12075190)

)

results_table1 <- apply_signal_function(appendixC_table1)

print(results_table1)

write.csv(results_table1, "results_appendixC_table1.csv", row.names = FALSE)

**8. Code for Supplementary Appendix C Table 2 (Indication-Specific Analyses; reproduces Table 6 and Supplementary Table 3)**

appendixC_table2 <- data.frame(

Drug = c(rep("Adalimumab", 5), rep("Etanercept", 5)),

Indication = c(

"Psoriasis", "Crohn's disease", "Rheumatoid arthritis",

"Ankylosing spondylitis", "Product used for unknown indication",

"Rheumatoid arthritis", "Psoriasis", "Ankylosing spondylitis",

"Drug use for unknown indication", "Product used for unknown indication"

),

a = c(48,33,24,19,8, 37,31,21,1,15),

b = c(27585,26137,18473,7320,3702, 33339,31126,8696,106,11054),

c = c(101,116,125,130,141, 71,77,87,107,93),

d = c(70081,71529,79193,90346,93964, 54836,57049,79479,88069,77121)

)

results_table2 <- apply_signal_function(appendixC_table2)

print(results_table2)

write.csv(results_table2, "results_appendixC_table2.csv", row.names = FALSE)

**9. Code for Supplementary Appendix C Table 3 (PT-Specific Analyses; reproduces Table 7 and Supplementary Table 4)**

appendixC_table3 <- data.frame(

Drug = c(

rep("Adalimumab", 7), rep("Etanercept", 6),

rep("Adalimumab", 4), rep("Etanercept", 3)

),

Indication = c(

rep("Psoriasis", 13),

rep("Ankylosing spondylitis", 7)

),

PT = c(

"Erectile dysfunction", "Libido decreased/loss", "Sexual dysfunction",

"Male sexual dysfunction", "Dyspareunia", "Ejaculation disorder",

"Disturbance in sexual arousal",

"Erectile dysfunction", "Sexual dysfunction", "Libido decreased/loss",

"Ejaculation disorder", "Disturbance in sexual arousal",

"Male sexual dysfunction",

"Erectile dysfunction", "Libido decreased/loss", "Sexual dysfunction",

"Ejaculation disorder",

"Erectile dysfunction", "Sexual dysfunction", "Libido decreased/loss"

),

a = c(37,7,5,1,1,1,1, 16,7,8,3,1,1, 10,8,5,1, 18,5,5),

b = c(91582,91612,91614,91618,91618,91618,91618,

84808,84817,84816,84821,84823,84823,

28908,28910,28913,28917,

27223,27236,27236),

c = c(131,37,48,2,0,3,1, 152,46,29,4,1,2, 44,10,15,2, 36,15,8),

d = c(324664,324758,324747,324793,324795,324792,324794,

331457,331563,331580,331605,331608,331607,

78231,78265,78260,78273,

79916,79937,79944)

)

results_table3 <- apply_signal_function(appendixC_table3)

print(results_table3)

write.csv(results_table3, "results_appendixC_table3.csv", row.names = FALSE)

**10. Optional Code for Direct Adalimumab-versus-Etanercept ROR Comparison in Common Indications**

ror_ratio_ci <- function(ror1, se1, ror2, se2) {

ratio <- ror1 / ror2

se_log_ratio <- sqrt(se1^2 + se2^2)

lower <- exp(log(ratio) - 1.96 * se_log_ratio)

upper <- exp(log(ratio) + 1.96 * se_log_ratio)

data.frame(ROR_ratio = ratio, lower95 = lower, upper95 = upper)

}

\# Example: ankylosing spondylitis

ada_as <- calc_signal(19, 7320, 130, 90346)

eta_as <- calc_signal(21, 8696, 87, 79479)

se_ada_as <- sqrt(1/19 + 1/7320 + 1/130 + 1/90346)

se_eta_as <- sqrt(1/21 + 1/8696 + 1/87 + 1/79479)

ror_ratio_ci(ada_as$ROR, se_ada_as, eta_as$ROR, se_eta_as)

**11. Verification Examples**

**Etanercept, ankylosing spondylitis (Appendix C Table 2):** a = 21, b = 8,696, c = 87, d = 79,479. The code reproduces ROR ≈ 2.21, PRR ≈ 2.20, E(IC) ≈ 0.90, IC025 ≈ 0.23, EBGM ≈ 1.97, and EBGM05 ≈ 1.32.

**Etanercept–erectile dysfunction within ankylosing spondylitis (Appendix C Table 3):** a = 18, b = 27,223, c = 36, d = 79,916. The code reproduces ROR ≈ 1.47, PRR ≈ 1.47, IC025 ≈ −0.42, and EBGM05 ≈ 0.82.

**Note on zero cells.** For rows containing a zero comparator cell, uncorrected ROR/PRR and EBGM05 are returned as not estimable (NA), consistent with the prespecified analysis and the notes in the supplementary tables. BCPNN remains estimable because of the specified Bayesian prior parameters.
