# Supplementary material for: Male sexual dysfunction associated with TNF-α inhibitors: an indication-stratified global pharmacovigilance analysis
Source: Front Pharmacol. 2026 Sep 14;17:1915246. doi: 10.3389/fphar.2026.1915246 (PMC13617334; doi:10.3389/fphar.2026.1915246)
Supplement: Supplementary file 2 [file Table1.docx]

**Supplementary Table 1. Classification and Terminology of Male Sexual Dysfunction**

| **HLGT** | **HLT** | **SOC** | **PT** |
| --- | --- | --- | --- |
| Sexual Function And Fertility Disorders | Erection And Ejaculation Conditions and Disorders | Reproductive System and Breast Disorders | Erectile Dysfunction |
|  |  |  | Organic Erectile Dysfunction |
|  |  |  | Ejaculation Disorder |
|  |  |  | Ejaculation Failure |
|  |  |  | Retrograde Ejaculation |
|  |  |  | Spontaneous Ejaculation |
|  |  |  |  |
|  | Sexual Function and Fertility Disorders | Reproductive System and Breast Disorders | Dyspareunia |
|  |  |  | Male Sexual Dysfunction |
|  |  |  | Sexual Dysfunction |
|  |  |  |  |
| Sexual Dysfunctions, Disturbances and Gender Identity Disorders | Orgasmic Disorders and Disturbances | Psychiatric Disorders | Premature Ejaculation |
|  |  |  | Male Orgasmic Disorder |
|  |  |  | Orgasm Abnormal |
|  |  |  | Orgasmic Sensation Decreased |
|  | Sexual Arousal Disorders | Psychiatric Disorders | Psychogenic Erectile Dysfunction |
|  |  |  | Disturbance In Sexual Arousal |
|  |  |  | Sexual Inhibition |
|  | Sexual Desire Disorders | Psychiatric Disorders | Libido Decreased |
|  |  |  | Libido Disorder |
|  |  |  | Loss Of Libido |

**Abbreviations:** SOC, *System Organ Class*; HLGT, *High Level Group Term*; HLT, *High Level Term*; PT, *Preferred Term* — all terms are defined according to the MedDRA (Medical Dictionary for Regulatory Activities) hierarchy.

**Supplementary Table 2. 2 × 2 contingency table for disproportionality analysis.**

|  | **Target AE** | **Non-target AE** |
| --- | --- | --- |
| **Drug of interest** | a | b |
| **All other drugs / comparison background** | c | d |

a represents the number of reports of the target event associated with the drug of interest;

b represents the number of reports of all other (non-target) events for the same drug;

c and d represent the corresponding counts for all other drugs in the database.

**Supplementary Table 3. Indication-stratified disproportionality analyses of male sexual dysfunction associated with adalimumab and etanercept using four predefined signal detection algorithms.**

| **Drug** | **Indication** | **a** | **ROR** | **ROR 95% CI** | **PRR** | **PRR 95% CI** | **E(IC)** | **IC025** | **EBGM** | **EBGM05** |
| --- | --- | --- | --- | --- | --- | --- | --- | --- | --- | --- |
| **Adalimumab** | Psoriasis | 48 | 1.21 | 0.86–1.70 | 1.21 | 0.86–1.70 | 0.18 | -0.30 | 1.14 | 0.86 |
|  | Crohn's disease | 33 | 0.78 | 0.53–1.15 | 0.78 | 0.53–1.15 | -0.27 | -0.82 | 0.83 | 0.60 |
|  | Rheumatoid arthritis | 24 | 0.82 | 0.53–1.27 | 0.82 | 0.53–1.27 | -0.23 | -0.86 | 0.85 | 0.59 |
|  | Ankylosing spondylitis | 19 | 1.80 | 1.11–2.92 | 1.80 | 1.11–2.91 | 0.71 | 0.02 | 1.70 | 1.14 |
|  | Product used for unknown indication | 8 | 1.44 | 0.71–2.94 | 1.44 | 0.71–2.93 | 0.43 | -0.56 | 1.42 | 0.78 |
| **Etanercept** | Rheumatoid arthritis | 37 | 0.86 | 0.58–1.28 | 0.86 | 0.58–1.28 | -0.15 | -0.70 | 0.91 | 0.65 |
|  | Psoriasis | 31 | 0.74 | 0.49–1.12 | 0.74 | 0.49–1.12 | -0.30 | -0.88 | 0.81 | 0.57 |
|  | Ankylosing spondylitis | 21 | 2.21 | 1.37–3.55 | 2.20 | 1.37–3.55 | 0.90 | 0.23 | 1.97 | 1.32 |
|  | Product used for unknown indication | 15 | 1.13 | 0.65–1.94 | 1.13 | 0.65–1.94 | 0.13 | -0.65 | 1.11 | 0.70 |
|  | Drug use for unknown indication | 1 | 7.76 | 1.07–56.14 | 7.70 | 1.08–54.68 | 0.82 | -1.26 | 7.64 | 1.46 |

**Abbreviations**: ROR, reporting odds ratio; PRR, proportional reporting ratio; BCPNN, Bayesian confidence propagation neural network; MGPS, multi-item gamma Poisson shrinker; CI, confidence interval; IC, information component; IC025, lower limit of the 95% credibility interval of the information component; EBGM, empirical Bayes geometric mean; EBGM05, lower one-sided 95% confidence limit of the EBGM.

**Signal detection criteria:** ROR: a ≥ 3 and lower 95% CI > 1; PRR: a ≥ 3 and lower 95% CI > 1; BCPNN: a ≥ 3 and IC025 > 0; MGPS: a > 0 and EBGM05 > 2.0.

**Supplementary Table 4. Preferred-term-stratified disproportionality analyses of male sexual dysfunction associated with adalimumab and etanercept**

| **Drug** | **Indication** | **Preferred term** | **Reports (a)** | **ROR (95% CI)** | **PRR (95% CI)** | **IC025** | **EBGM05** |
| --- | --- | --- | --- | --- | --- | --- | --- |
| Adalimumab | Psoriasis | Erectile dysfunction | 37 | 1.00 (0.70-1.44) | 1.00 (0.70-1.44) | -0.53 | 0.74 |
|  |  | Libido decreased/loss | 7 | 0.67 (0.30-1.50) | 0.67 (0.30-1.50) | -1.55 | 0.37 |
|  |  | Sexual dysfunction | 5 | 0.37 (0.15-0.93) | 0.37 (0.15-0.93) | -2.34 | 0.20 |
|  |  | Male sexual dysfunction | 1 | 1.77 (0.16-19.55) | 1.77 (0.16-19.55) | -2.41 | 0.20 |
|  |  | Dyspareunia | 1 | Not estimable | Not estimable | -2.41 | Not estimable |
|  |  | Ejaculation disorder | 1 | 1.18 (0.12-11.36) | 1.18 (0.12-11.36) | -2.48 | 0.17 |
|  |  | Disturbance in sexual arousal | 1 | 3.55 (0.22-56.68) | 3.55 (0.22-56.68) | -2.37 | 0.22 |
| Etanercept | Psoriasis | Erectile dysfunction | 16 | 0.41 (0.25-0.69) | 0.41 (0.25-0.69) | -1.79 | 0.30 |
|  |  | Sexual dysfunction | 7 | 0.59 (0.27-1.32) | 0.59 (0.27-1.32) | -1.68 | 0.33 |
|  |  | Libido decreased/loss | 8 | 1.08 (0.49-2.36) | 1.08 (0.49-2.36) | -1.03 | 0.55 |
|  |  | Ejaculation disorder | 3 | 2.93 (0.66-13.10) | 2.93 (0.66-13.10) | -1.16 | 0.60 |
|  |  | Disturbance in sexual arousal | 1 | 3.91 (0.24-62.51) | 3.91 (0.24-62.50) | -2.32 | 0.24 |
|  |  | Male sexual dysfunction | 1 | 1.95 (0.18-21.56) | 1.95 (0.18-21.56) | -2.36 | 0.22 |
| Adalimumab | Ankylosing spondylitis | Erectile dysfunction | 10 | 0.62 (0.31-1.22) | 0.62 (0.31-1.22) | -1.48 | 0.39 |
|  |  | Libido decreased/loss | 8 | 2.17 (0.85-5.49) | 2.17 (0.85-5.49) | -0.61 | 0.76 |
|  |  | Sexual dysfunction | 5 | 0.90 (0.33-2.48) | 0.90 (0.33-2.48) | -1.49 | 0.40 |
|  |  | Ejaculation disorder | 1 | 1.35 (0.12-14.93) | 1.35 (0.12-14.93) | -2.55 | 0.17 |
| Etanercept | Ankylosing spondylitis | Erectile dysfunction | 18 | 1.47 (0.83-2.59) | 1.47 (0.83-2.58) | -0.42 | 0.82 |
|  |  | Sexual dysfunction | 5 | 0.98 (0.36-2.69) | 0.98 (0.36-2.69) | -1.41 | 0.42 |
|  |  | Libido decreased/loss | 5 | 1.83 (0.60-5.61) | 1.83 (0.60-5.61) | -1.01 | 0.59 |

**Abbreviations:** ROR, reporting odds ratio; PRR, proportional reporting ratio; BCPNN, Bayesian confidence propagation neural network; MGPS, multi-item gamma Poisson shrinker; CI, confidence interval; IC025, lower limit of the 95% credibility interval of the information component; EBGM05, lower one-sided 95% confidence limit of the empirical Bayes geometric mean.

**Signal detection criteria: ROR: a ≥ 3 and lower 95% CI > 1; PRR: a ≥ 3 and lower 95% CI > 1; BCPNN: a ≥ 3 and IC025 > 0; MGPS: a > 0 and EBGM05 > 2.0.**

**Note:** All estimates were recalculated from the same corrected 2 × 2 contingency tables used for Table 7. Complete underlying cell counts (a, b, c, and d) are provided in Supplementary Appendix C. For rows containing a zero-comparator cell, ROR/PRR and EBGM05 were not estimable using the prespecified uncorrected formulas.

Supplementary Table 5. Univariate logistic regression analysis of age associated with target preferred terms in the Adalimumab cohort

| **Variable** | **Category** | **OR** | **95% CI** | **P value** |
| --- | --- | --- | --- | --- |
| **Age (years)** | 18–64.9 | Reference | — | — |
|  | ≥65 | 0.37 | 0.18–0.73 | 0.004 |

Abbreviations: CI = Confidence Interval, OR = Odds Ratio.

Supplementary Table 6. Univariate logistic regression analysis of age associated with target preferred terms in the Etanercept cohort

| Variable | Category | OR | 95% CI | P value |
| --- | --- | --- | --- | --- |
| Age (years) | 18–64.9 | Reference | — | — |
|  | ≥65 | 0.35 | 0.19–0.66 | 0.001 |
|  |  |  |  |  |

Abbreviations: CI = Confidence Interval, OR = Odds Ratio.
